# Antidepressant Treatment for Acute Bipolar Depression: An Update

**DOI:** 10.1155/2012/684725

**Published:** 2012-01-27

**Authors:** Ben H. Amit, Abraham Weizman

**Affiliations:** ^1^Geha Mental Health Center, Research Unit, P.O. Box 102, Petach Tikva 49100, Israel; ^2^Sackler Faculty of Medicine, Tel Aviv University, P.O. Box 39040, Tel Aviv 69978, Israel

## Abstract

While studies in the past have focused more on treatment of the manic phase of bipolar disorder (BD), recent findings demonstrate the depressive phase to be at least as debilitating. However, in contrast to unipolar depression, depression in bipolar patients exhibits a varying response to antidepressants, raising questions regarding their efficacy and tolerability. *Methods*. We conducted a MEDLINE and Cochrane Collaboration Library search for papers published between 2005 and 2011 on the subject of antidepressant treatment of bipolar depression. Sixty-eight articles were included in the present review. *Results*. While a few studies did advocate the use of antidepressants, most well-controlled studies failed to show a robust effect of antidepressants in bipolar depression, regardless of antidepressant class or bipolar subtype. There was no significant increase in the rate of manic/hypomanic switch, especially with concurrent use of mood stabilizers. Prescribing guidelines published in recent years rely more on atypical antipsychotics, especially quetiapine, as a first-line therapy. *Conclusions*. Antidepressants probably have no substantial role in acute bipolar depression. However, in light of conflicting results between studies, more well-designed trials are warranted.

## 1. Introduction

Bipolar disorder (BD) is a devastating illness, carrying an immense burden of both morbidity [1] and all-cause mortality [2], including high rates of completed suicide [3]. With a lifetime prevalence of 1.5–2% in Europe [4] and a similar prevalence in the USA [5], much attention has been drawn to assessing potential treatments for alleviating the symptoms of this condition, manic and depressive alike. However, while clinical focus in the past tended to be more on the manic phase of the disorder, recent findings illustrate the need to focus on effective treatment strategies for the depressive phase, for several reasons. First, observations of the natural course of BD show the considerable amount of time spent in the depressive phase compared to the manic phase (30% on average compared to 10% in bipolar 1 disorder) [6], leading to severe morbidity, including a marked occupational impairment [7]. Second, the depressive phase of BD is more prone to suicide [8]. Incomplete remission, with enduring subsyndromal depressive symptoms, has been demonstrated both to cause functional impairment [9] and increase the risk of relapse [10], emphasizing the importance of optimizing the treatment for the depressive phase of BD.

Since their conception, antidepressants have been the mainstay of treatment for depression of any kind; to this day, antidepressants are prescribed to patients suffering from bipolar depression in 50% of cases [11]. However, observations regarding questionable efficacy and tolerability of antidepressants in bipolar depression have prompted a huge debate on the topic in recent years. We, therefore, wished to review the latest articles addressing the role of antidepressants in the pharmacological treatment of acute bipolar depression.

## 2. Methods

We conducted a MEDLINE and Cochrane Collaboration Library search for any English-language articles published from January 1st 2005 to August 31st 2011, containing the keywords “treatment,” “bipolar,” and “depression” in the title or abstract. The search included clinical trials, meta-analyses, review articles, practice guidelines, conference summaries, editorials, and comments. We further used the “related citation” property for each item to search for other pertinent articles.

Initial screening yielded 1,062 results. Abstracts were then reviewed to exclude articles of low relevance, such as those regarding treatments other than antidepressants or concerning patients whose age is above 65 or below 18 years. A total of 68 items were finally included in the review.

## 3. Results

### 3.1. Antidepressant Monotherapy

#### 3.1.1. Antidepressant Monotherapy for Bipolar I Disorder

**Fluoxetine**

In light of evidence of an increased risk of manic induction resulting from unopposed antidepressant treatment in bipolar I disorder [12], there has been a scarcity of studies in recent years examining the role of antidepressant monotherapy in bipolar I depression. In a 2005 randomized clinical trial consisting of 34 bipolar patients, 32 of which of the bipolar I subtype, treatment with 10–30 mg of fluoxetine showed comparable results to treatment with either olanzapine or an olanzapine-fluoxetine combination. Over the course of the 8-week trial, a significant reduction in both HAM-D 28 and MADRS ratings was observed, with no evidence of an increase in treatment-emergent manic symptoms, as measured by the Young Mania Rating Scale (YMRS) [13].

**Paroxetine**

In the 2010 EMBOLDEN II study, a total of 740 depressed bipolar patients (478 bipolar I, 262 bipolar II) were treated by monotherapy with either paroxetine (20 mg/d), quetiapine (300 mg/d or 600 mg/d), or placebo. An eight-week followup revealed no statistically significant change in MADRS total score for paroxetine compared with placebo, both in bipolar I and bipolar II patients, in contrast with a marked response observed in the quetiapine arm. Manic/hypomanic switch rates, defined as Young Mania Rating Scale (YMRS) ≥16, did not statistically differ between paroxetine and placebo (10.7% and 8.9%, resp.) [14], rendering paroxetine a safe, yet not an efficacious, option as monotherapy for bipolar depression.

#### 3.1.2. Antidepressant Monotherapy for Bipolar II Disorder

**Fluoxetine**

Concerns about the risk of manic induction have prompted studies of antidepressant monotherapy in recent years to focus on bipolar II patients, where the risk has been estimated to be lower. One large study examining the response to antidepressant monotherapy in BP II patients included a 14-week open-label trial of 148 patients, treated by 10–80 mg fluoxetine daily. Response rate was demonstrated to be 59.5% (95% CI, 51.1%–67.4%, *P* < 0.0005) and remission rate 58.1% (95% CI, 49.7%–66.2%, *P* < 0.0005). 4.1% of patients had treatment-emergent hypomania, defined as YMRS score of 8 or greater (95% CI, 1.5%–8.6%, *P* < 0.0005), while 2.7% (95% CI, 0.7%–6.8%, *P* < 0.0005) had a YMRS score of 12 or greater. 19.6% of patients had subsyndromal hypomania (95% CI, 13.5%–26.9%, *P* < 0.0005), defined as an episode lasting 3 or less days with 4 symptoms or more, or as an episode lasting 4 days or more with 3 symptoms or less. Although one patient had treatment-emergent mania, reexamination of his medical record revealed a diagnosis of BP I disorder, rather than BP II. The authors concluded by deeming fluoxetine monotherapy a safe and effective short-term treatment of bipolar II depression, with a relatively low syndromal mood conversion rate [15].

**Escitalopram**

In a small, randomized, placebo-controlled proof of concept study (*n* = 10), treatment with escitalopram demonstrated a significant improvement in the depressive symptoms and functioning status of BPII patients over nine months, with no evidence of an affect switch, leading the author to suggest SSRIs as “mood stabilizers for Bipolar II Disorder” [16].

**Venlafaxine**

In a randomized open-label clinical trial including 83 BPII patients, 43 were randomized to treatment with venlafaxine and 40 to lithium monotherapy. Following a 12-week observation period, venlafaxine surpassed lithium both in response rates (58.1% versus 20.0%; *P* < 0.0005) and in remission rates (44.2% versus 7.5%; *P* < 0.0005), with no significant increase in mean YMRS scores [17]. A secondary analysis of the data showed no difference in treatment response between rapid and nonrapid cyclers [18]. Switch to venlafaxine treatment for lithium nonresponders resulted in a significant improvement in depressive symptoms, with no evidence of manic induction over a follow-up period of 12 weeks [19]. Another smaller study of fifteen depressed female patients with a diagnosis of BPII disorder corroborated these findings, demonstrating no episodes of drug-induced mania or hypomania during 6 weeks of venlafaxine monotherapy [20].

**Tricyclic Antidepressants and Monoamine Oxidase Inhibitors**

In a 2007 randomized controlled trial, 70 BP II patients were treated with the tricyclic antidepressant imipramine (average dose 250 mg/d) or the monoamine oxidase inhibitor phenelzine (average dose 60 mg/d), showing a response rate of 57% and 52%, respectively, compared with 23% in the placebo arm. Data regarding statistical significance was lacking. Although there was no evidence of manic induction [21], these results are limited, as no valid tool was used to assess treatment-emergent manic/hypomanic symptoms.

### 3.2. Antidepressants as Adjuncts to Mood Stabilizers

#### 3.2.1. Efficacy and Tolerability

In a meta-analysis of 12 trials encompassing a total of 1,088 patients, published in 2004 by Gijsman et al. [22], antidepressants of the selective serotonin reuptake inhibitor class (SSRIs), tricyclic antidepressants (TCAs), and monoamine oxidase inhibitors (MAOIs) were demonstrated to be effective as adjuncts to mood stabilizers in the treatment of acute bipolar depression. Analysis of four randomized controlled trials, consisting of 662 patients, most of them treated by concurrent mood stabilizers, has shown a significant advantage in achieving response for the group treated with an antidepressant (fluoxetine, imipramine, or the MAOIs tranylcypromine and selegiline) compared to placebo (risk ratio = 1.86, 95% CI = 1.49–2.30), with a number needed to treat (NNT) of 4.2 (95% CI = 3.2–6.4). Patients treated with an antidepressant (paroxetine, imipramine, or fluoxetine) were also more likely to reach remission than those who were not taking an antidepressant (risk ratio = 1.41, 95% CI = 1.11–1.80), with an NNT of 8.4 (95% CI = 4.8–33). The risk of manic switch following the use of SSRIs was 3.2%, not significantly greater than placebo; however, the authors stated that the low incidence of manic events over a short follow-up period of four to ten weeks limits the power to detect a significant difference. The rate of manic switch following the use of TCAs was demonstrated to be as high as 10%, an absolute risk difference of 6.8% (95% CI = 1.7%–11.9%); however, no valid scales were used to assess manic symptoms, causing a problem with data interpretation (see Section 3).

The authors concluded that SSRIs may be an effective treatment for acute bipolar depression, with a low risk of manic switch early in the course of treatment.

Although the recommendation to use antidepressants as adjuncts to mood stabilizers in the treatment of acute bipolar depression did not conflict with common practices at the time, as reflected in the 2003 British Association for Psychopharmacology guideline [23], it was “at odds” with the American Psychiatric Association (APA) guideline, published in April 2002, where lithium or lamotrigine was recommended as a first-line agent [24]. The meta-analysis received many comments, concerning the short duration of studies [25], the heterogeneous inclusion of patients with bipolar II depression or mixed episodes, and the concomitant use of a mood stabilizer in the majority of patients, allegedly responsible for the low rate of manic switch observed [26].

Over the course of the next few years, numerous trials and meta-analyses sought to clarify the question of the role of antidepressants as adjuncts to mood stabilizers in the acute treatment of bipolar depression. One small double-blind randomized trial (*n* = 20) compared the addition of lamotrigine versus citalopram to treatment with a mood stabilizer in bipolar depressed patients; though both treatments demonstrated a significant decrease in MADRS scores after six weeks of treatment with no evidence of major adverse events, the lack of a placebo arm and the small sample size limit the applicability of the data [27]. A similar size open-label trial of 12-week addition of escitalopram to treatment with mood stabilizer showed comparable results, including a mean decrease in HAM-D score of 12 points (*P* < .001). Although three cases of mania/hypomania were described, the small sample size and lack of control represent a problem with data interpretation [28].

Perhaps one of the most quoted studies on the topic is the 2007 trial conducted by the Systematic Treatment Enhancement Program for Bipolar Disorder (STEP-BD) collaborators, published in the New England Journal of Medicine by Sachs et al. [29]. In this double-blinded, randomized controlled trial of 366 Bipolar I and II patients, subjects receiving treatment with a mood stabilizer were randomized to cotreatment with an SSRI antidepressant, either bupropion or paroxetine. The duration of followup was 26 weeks, and the primary outcome was defined as at least 8 consecutive weeks of euthymia. However, contrary to the findings presented by Gijsman et al. [22], the study did not find a significant effect of either SSRI in any parameter, including remission or response. The reason for this disparity, as suggested by the authors, may lie in the more naturalistic design of the trial, allowing inclusion of patients with various comorbidities, such as anxiety disorders, substance abuse, or psychotic symptoms, as well as those receiving additional pharmacotherapy or psychotherapy [29]. As was the case in the previous meta-analysis by Gijsman et al. [22], SSRIs did not show an increased risk of manic switch when coadministered with a mood stabilizer. However, further retrospective analysis of the risk of a manic switch, based on self-report, did reveal an increased risk of switch, correlating with a shorter duration of illness and a history of multiple antidepressant trials [30]. In a different trial assessing the risk of switch, bipolar subtype was also demonstrated to correlate with the risk of treatment-emergent mania/hypomania, with patients diagnosed with the bipolar II subtype showing significantly less susceptibility to switch (12% and 2%, resp.; YMRS > 14) [31].

In a recent meta-analysis by Sidor and MacQueen [32], six trials comparing antidepressants to placebo in the acute (4–16 weeks) treatment of depressed bipolar I or II patients were analyzed [13, 29, 33–37]; 68% of patients were treated by concomitant mood stabilizers. Although the effect of antidepressants was nonsignificant compared to placebo in induction of clinical response (95% CI 0.99–1.40; *P* = .06), the authors pointed out the heterogeneity of the studies, which was assigned to the largest trial—published by Sachs et al. [29]—showing a negative treatment effect, favoring placebo. Analyzing for clinical remission showed similar results, also failing to show a significant benefit of antidepressants over placebo (95% CI 0.98–1.47; *P* = .09). Rates of switch to mania/hypomania by using a Young Mania Rating Scale (YMRS) threshold of 12 were 7.7% for antidepressants and 7.2% for placebo, a non-significant difference (RR = 0.97; 95% CI 0.62–1.53; *P* = .90). When discussing the discordance between the results of this meta-analysis and the one published by Gijsman et al. [22], the authors stated that two of the four trials used in the aforementioned analysis, favoring antidepressants (published in 1982 and 1980 by Himmelhoch et al. [38] and Mendlewicz and Youdim [39], resp.), did not properly differentiate between bipolar and unipolar depression, causing marked bias towards antidepressant efficacy [32].

#### 3.2.2. Comparison of Drug Classes: SSRIs, SNRIs, and TCAs

In addition to the general question regarding the efficacy and tolerability of antidepressants in the treatment of bipolar depression, several studies in recent years have attempted to compare the individual properties of various antidepressants. Among them is the 2006 publication by Post et al. [40], comparing a randomized addition of venlafaxine, sertraline, or bupropion to maintenance treatment with lithium during treatment-emergent depression. Of the 174 patients enrolled in the study, 49–53% demonstrated a response to treatment while 34–41% reached remission after 10 weeks, using the Inventory of Depressive Symptomatology (IDS) and Clinical Global Impression for Bipolar Disorder (CGI-BP) scales. There was no significant difference in efficacy between drug classes. Manic switch, using a YMRS threshold score of 13, ensued in 4% of patients on bupropion, 7% of patients on sertraline, and 15% on venlafaxine, with a nonsignificant trend towards venlafaxine being more harmful (*P* = 0.052). Adding a more liberal criterion of manic switch, such as a CGI-BP severity of mania ≥3, yielded a significant difference between drug classes, with venlafaxine showing a higher risk of switch than both sertraline or bupropion (*P* = 0.03); however, the lack of a placebo arm seriously under-powers this study. Interestingly, the higher risk of manic switch in the venlafaxine group was accounted for by the rapid-cycling subset of patients, which constituted 27% of the sample, showing particular sensitivity to manic switch following venlafaxine treatment.

Another recent comparison of antidepressant classes as adjuncts to mood stabilizers is the 2010 randomized clinical trial published by Pilhatsch et al. [41]. Forty depressed bipolar I and II patients, on maintenance treatment with lithium, were randomized to receive either adjunctive paroxetine or amitriptyline. Following a six-week follow-up period, both treatments were shown to be equally as effective, with no significant difference in HAM-D reduction (−14.9 versus −15.5; *P* = 0.798) or final HAM-D21 score (8.2 versus 9.9; *P* = 0.420) between paroxetine and amitriptyline, respectively. Treatment with paroxetine did show a significantly more rapid onset, evident since the third week of treatment. While one patient treated with paroxetine had treatment emergent hypomania, fewer adverse events were recorded for the group treated with paroxetine than for amitriptyline, with an emergent symptom index of 4.1 and 5.0 per patient in each group, respectively, making paroxetine a better overall first choice of the two.

A more intricate look into the potential use of antidepressants as adjuncts to treatment is a comparative trial employing lithium, lamotrigine, and paroxetine in two different treatment algorithms [42]. 124 depressed bipolar patients receiving maintenance treatment with lithium were randomly assigned to additional treatment with either lamotrigine or placebo. After eight weeks, nonresponders were treated with supplementary paroxetine 20 mg/d. While addition of lamotrigine proved effective compared to placebo at week 8, adding paroxetine to nonresponders “blunted” this effect, causing the two groups to demonstrate no significant differences in MADRS score by week 16. While this might indicate paroxetine as a potential efficacious agent, the lack of a second placebo arm, controlling for the effect of paroxetine, causes an inability to rule out at least some degree of spontaneous recovery, unrelated to paroxetine use.

#### 3.2.3. Olanzapine/Fluoxetine Combination

Since the introduction of Symbyax, an olanzapine/fluoxetine combination (OFC), it has gained widespread use for the treatment of bipolar depression, becoming the first treatment to be approved by the U.S. Federal Drug Administration for this indication in 2003 [43]. In a double-blind, 8-week, randomized controlled trial published by the Lilly Research Laboratories the same year, 833 adult bipolar I patients were randomized to treatment with olanzapine, OFC, or placebo. Both treatments were significantly (*P* < 0.001) more effective than placebo in reducing MADRS scores, with corresponding therapeutic effect sizes for olanzapine and OFC of 0.32 and 0.68, respectively. OFC proved significantly better than olanzapine alone as of week 4, meeting remission criteria by the end of the 8-week trial in 48.8% of cases. No major adverse events were reported, including treatment-emergent mania/hypomania [33, 34]. Health-related quality of life was also demonstrated to improve [44]. In another randomized comparative study of 34 bipolar I and II patients, discussed earlier, olanzapine/fluoxetine combination showed a significant decrease in both HAM-D 28 and MADRS ratings compared to placebo, with no evidence of a significant increase in manic symptoms [13]. An additional Lilly Research Laboratories publication from 2006 compared OFC to lamotrigine in the acute, 7-week treatment of 410 bipolar I patients. While OFC demonstrated a significant advantage over lamotrigine in reducing MADRS scores, the overall effect size was relatively small (*P* = .002, effect size = 0.24). Though time to response was significantly shorter for OFC-treated patients (OFC median days = 17 versus lamotrigine 23; *P* = .01), the response rates—albeit high (OFC, 68.8% versus LMG, 59.7%; *P* = .073)—did not significantly differ between both treatment groups. In addition, OFC-treated patients suffered significantly more adverse events, including sedation, weight gain, and tremor, as well as having increased levels of total cholesterol and triglycerides. Interestingly, genotyping of SNPs within the dopamine D3 receptor and histamine H1 receptor genes was significantly associated with response to OFC, possibly demonstrating the importance of the dopaminergic system in the treatment of patients with bipolar I depression [45].

Another study worth mentioning in this context is a Lilly-sponsored open-label continuation trial of 114 bipolar patients in Puerto Rico. The first phase of the trial included a 7-week treatment course with OFC, demonstrating a response rate of 69% and a remission rate of 59%, in accordance with earlier findings. Responders were then randomized to either OFC continuation or olanzapine alone for 12 weeks, showing maintenance of response to be significantly higher for the OFC group than the olanzapine group (31.3% versus 12.5%). Metabolic adverse effects were highly prevalent, with 33% of the OFC-treated patients gaining over 7% of their body weight over the 4-month course [46].

## 4. Discussion

It is astounding how, despite numerous trials and meta-analyses conducted on the subject in recent years, the role of antidepressants in the treatment of bipolar depression still remains unclear. Since the 2004 meta-analysis by Gijsman et al. [22], demonstrating antidepressants as a whole, and SSRIs in particular, to be both effective and safe as an add-on therapy for acute bipolar depression, many studies published since showed evidence both supporting and contradicting these results.

### 4.1. Antidepressant Efficacy

A marked disparity exists between the results of studies examining the efficacy of antidepressants in acute bipolar depression, whether as monotherapy or as an adjunct to mood stabilizers (Table 1). Although as a whole more studies concluded in favor of antidepressant treatment efficacy in both modalities, most of them suffered major methodological disadvantages, such as lack of a placebo arm [13, 15, 17, 27, 28, 41], small sample size [16, 27, 28], or substantial industry involvement [44–46]. However, although industry-sponsored, it is hard to dismiss the significant efficacy demonstrated for the first FDA-approved therapy for bipolar depression, olanzapine/fluoxetine combination (OFC), showing an effect size of 0.68 compared to 0.32 of olanzapine alone [33, 34]. On the other hand, the two studies showing lack of antidepressant efficacy were based on results of the STEP-BD [29] and EMBOLDEN II trials [14], both of high methodological quality in terms of randomization, control, blinding, and sample size. Thus, a more recent meta-analysis, published in 2011 and incorporating the results of recent trials, showed no significant efficacy of antidepressants in the treatment of acute bipolar depression [32]. In an attempt to address the discrepancy between the positive effects of antidepressants demonstrated by Gijsman et al. [22] and more recent results, the authors pointed out to potential flaws in the analysis, among which is the inclusion of studies causing bias for antidepressant efficacy [32]. However, several issues need to be taken into account, prior to regarding antidepressants as ineffective for this indication. First is the fact that—although not statistically significant—antidepressant efficacy in the more recent meta-analysis was *very close* to demonstrating significance in both induction of remission (95% CI 0.99–1.40; *P* = .06) and response (95% CI 0.98–1.47; *P* = .09) [32]. Second is the fact that the largest negative trial incorporated in the meta-analysis, based on STEP-BD results [29], included a substantial negative treatment effect, favoring placebo over antidepressants. Although such data is unavailable, it is quite possible that correcting for this effect might have shifted the confidence interval in the meta-analysis slightly, rendering antidepressants significantly superior to placebo, even if not by much.

Finally, the heterogeneous inclusion of patients treated with various “mood stabilizers,” some of them possessing antidepressant activity, such as quetiapine [14] or olanzapine [33, 34], may cause an additional difficulty in interpreting the data; further trials of antidepressant augmentation of a more homogeneous “mood stabilizer” are advised.

### 4.2. The Risk of a Manic/Hypomanic Switch

The majority of recent studies do not demonstrate a significant risk of manic/hypomanic switch as a result of antidepressant treatment in acute bipolar depression, both as monotherapy and in conjunction with a mood stabilizer (Table 2). Only the use of very “liberal” criteria, such as self-report [30] or an interviewer impression of “subsyndromal hypomania” (defined as an episode lasting 3 or less days with 4 symptoms or more, or as an episode lasting 4 days or more with 3 symptoms or less) [15], has succeeded in providing evidence for an increased risk of switch as result of SSRI treatment. While a somewhat higher risk was associated with the use of the SNRI venlafaxine [40] or tricyclic antidepressants [22], data interpretation is difficult due to the lack of comparison to placebo [40], as well as the lack of use of objective clinical scales to assess an affective switch [22]. Indeed, considerable inconsistency exists between studies regarding the definition of such a switch, including the use of different scales, such as the Clinical Global Impression for Bipolar Disorder (CGI-BP) [40] or the Young Mania Rating Scale (YMRS), or using different cutoff scores for mania or hypomania on the scale, such as a YMRS score of 16 [14], 14 [31], 13 [40], 12 [29], or 8 [15]. The result is making data applicability to clinical practice difficult. However, while the overall risk of an affective switch during acute treatment with an antidepressant is probably low, especially with the use of SSRIs or bupropion, some patient populations have been identified as being more prone to this side effect, including those with the bipolar I subtype compared to bipolar II [31], rapid-cycling patients [40], and patients with a shorter duration of illness and a history of multiple antidepressant trials [30].

### 4.3. Therapeutic Implications

#### 4.3.1. Diagnosis

If antidepressants are indeed redundant in the treatment of bipolar depression, it should have a direct impact on several aspects of the care of depressed bipolar patients, as well as depressed patients in general. First is the heightened importance of reaching the correct initial diagnosis of bipolar depression, often misdiagnosed as unipolar depression upon first presentation [47]. While recent attempts have failed in providing unequivocal support for the role of misdiagnosed bipolar disorder as a major cause for refractory depression [48], clinicians have nevertheless been urged to be more sensitive to “soft” bipolar signs when making a diagnosis, including “lowering the threshold” for hypomanic episodes [49]. A timely diagnosis of bipolar disorder has been shown to correlate with better outcomes [50–52] as well as reduced healthcare costs [53, 54], illustrating the importance of making a correct diagnosis as early as possible.

#### 4.3.2. Assessment of Treatment Response

Aside from the importance of making a correct diagnosis, several other factors have been suggested as relevant in treating patients suffering from bipolar depression. One is the identification of clinical factors potentially associated with antidepressant resistance, including the severity of the current episode, presence of melancholic features, current suicidal risk, and psychiatric comorbidity, including social phobia [55]. Adequately assessing the presence of other comorbid conditions, such as substance use, is crucial, especially in light of the extensive effect they may have on treatment response [56] and the risk of affective switch [57].

Following initiation of treatment, assessment of response poses another challenge. In one large trial, early improvement of depressive symptomatology did not appear to be a reliable predictor of eventual response or remission due to an unacceptably high false-positive rate. However, the absence of early improvement appeared to be a highly reliable predictor of eventual nonresponse, demonstrating the need for close monitoring of patient status during the initial phase of treatment [58]. Adherence should also be closely monitored, as certain factors have been associated with nonadherence in bipolar disorder, including selected patient factors, such as demographic features, symptom severity and phase of illness, presence of past suicide attempts, psychiatric comorbidity, illness and treatment duration, and relationship with providers, as well as treatment factors, including type and intensity of treatment [59].

#### 4.3.3. Extent and Duration of Treatment

Another issue in the treatment of acute bipolar depression is the duration of treatment. In a trial conducted as part of the Systematic Treatment Enhancement Program for Bipolar Disorder (STEP-BD) study, patients responding to treatment with antidepressants plus mood stabilizers, and euthymic for 2 months, were randomly assigned to antidepressant continuation versus discontinuation. After a follow-up period of 1–3 years, antidepressant continuation showed a mildly delayed depressive episode relapse (HR = 2.13 [1.00–4.56]) and trended toward less severe depressive symptoms (mean difference = −1.84 [95% CI, −0.08 to 3.77]), without increased manic symptoms [60]. Other trials showed comparable results [61, 62], providing evidence to support recommendations for continuing long-term antidepressant treatment for responsive patients. However, the full consequences of long-term antidepressant treatment, including the potential of increasing the risk of an affective switch, are out of the scope of this discussion. The goal of treatment should be full remission, as subsyndromal depressive symptoms have been demonstrated to result in marked functional impairment [63].

In conclusion, it is worth noting that bipolar disorder is a complex condition, requiring a multimodal approach. The tools used in the treatment of bipolar disorder include various pharmacological treatments, including antidepressants, mood stabilizers, and antipsychotics, nonpharmacological treatment modalities, such as electroconvulsive therapy and transcranial magnetic stimulation, as well as psychotherapeutic approaches. Proper integration of all available modalities is necessary for optimal treatment response.

### 4.4. Impact on Prescribing Guidelines

Probably in light of the inconclusive nature of the evidence, a review of guidelines published in recent years has not revealed major changes in the recommendations regarding antidepressant treatment. While the American Psychiatric Association (APA) guideline for the treatment of bipolar disorder was not updated since 2005 [64], it did include a recommendation of olanzapine/fluoxetine combination as a first-line option in the treatment of bipolar depression. At the same time, evidence for the efficacy of an antidepressant with adjunctive mood stabilizer was described as modest, while prescription of antidepressants in the absence of a mood stabilizer was not recommended for bipolar I patients. In an International Consensus Group (ICG) updated in 2007 [65], antidepressant treatment for bipolar I depression was indicated only “as an acute adjunct to treatment, with no additional benefit in the long-term treatment of bipolar depression.” Evidence supporting efficacy in bipolar II depression has been assigned to the lowest category. The 2009 NICE guideline [66] does include a first-line treatment option with an antidepressant for moderate-severe depression, albeit with a concurrent antimanic agent only, as does the 2009 Canadian Network for Mood and Anxiety Treatments (CANMAT) guideline [67]. Recommendations of the British Association of Psychopharmacology (BAP) of the same year have been slightly more liberal, in allowing antidepressant monotherapy as a first-line option for patients with no past evidence of mania, yet with a recommendation for gradual discontinuation of treatment after 12 weeks [68].

In a 2010 update of the World Federation of Societies of Biological Psychiatry (WFSBP) guideline on the treatment of acute bipolar depression, only the olanzapine/fluoxetine combination has been approved as a first-line therapy for this indication. There were no additional recommendations regarding antidepressant therapy, aside for mentioning possible efficacy in bipolar II patients [69].

In summary, while the major trend in recent years has been the adoption of quetiapine monotherapy as a first-line agent, most guidelines still advocate the use of antidepressants as potential first-line agents in the acute treatment of bipolar depression, in adjunction to mood stabilizers. Specific references were made for the SSRIs paroxetine [67, 69] and sertraline [65, 69], the dopamine-norepinephrine reuptake inhibitor bupropion [65, 67, 69] and the serotonin- norepinephrine reuptake inhibitor venlafaxine [65, 67, 69].

## 5. Conclusions

Studies conducted in recent years have failed to demonstrate significant beneficial effects of antidepressants in the treatment of acute bipolar depression. The rate of manic/hypomanic switch is probably low, especially with concurrent use of mood stabilizers. However, the considerable disparity between studies should prompt further large-scale, long-term, double-blind, randomized clinical trials, including comparison between various classes of antidepressants.

## Figures and Tables

**Table 1:** Summary of recent studies examining the efficacy of antidepressants in the treatment of acute bipolar depression.

Positive studies				Negative studies			
Study	No. of participants	Drug	Comments	Study	No. of participants	Drug	Comments
*Antidepressants as monotherapy*							
Amsterdam and Shults 2005 [13]	34 (BPI = 32, BPII = 2)	Fluoxetine, olanzapine/fluoxetine combination	No placebo control	McElroy et al. 2010 (EMBOLDEN II) [14]	740 (BPI = 478, BPII = 262)	Paroxetine	
Parker et al. 2006 [16]	10 BPII	Escitalopram	Small sample size				
Agosti and Stewart 2007 [21]	70 BP II	Imipramine, phenelzine	No significance demonstrated				
Amsterdam and Shults 2008 [17]	83 BPII	Venlafaxine	No placebo control				
Amsterdam and Shults 2010 [15]	148 BPII	Fluoxetine	No placebo control				

*Antidepressants with mood stabilizers*							
Schaffer et al. 2006 [27]	20	Citalopram	Small sample size, no placebo control	Sachs et al. 2007 [29]	366 (BPI = 240, BPII = 114)	Paroxetine, bupropion	
Fonseca et al. 2006 [28]	20	Escitalopram	Small sample size, no placebo control	Sidor et al. 2011 [32]	1,034 (Meta- Analysis)	Fluoxetine, paroxetine, bupropion, imipramine	
Tamayo et al. 2009 [46]	114	Olanzapine/fluoxetine combination	Conducted by Lilly Research Laboratories				
Pilhatsch et al. 2010 [41]	40	Paroxetine, amitriptyline	No placebo control				
Perlis et al. 2010 [45]	410	Olanzapine/fluoxetine combination	Conducted by Lilly Research Laboratories				

**Table 2:** Summary of recent studies examining the risk of manic/hypomanic switch following the use of antidepressants in the treatment of acute bipolar depression.

Increased risk of switch				No increased risk of switch			
Study	No. of participants	Drug	Comments	Study	No. of participants	Drug	Comments
*Antidepressants as Monotherapy*							
				Amsterdam and Shults 2005 [13]	34 (BPI = 32, BPII = 2)	Fluoxetine, olanzapine/fluoxetine combination	No Placebo Control
				Parker et al. 2006 [16]	10 BPII	Escitalopram	Small sample size
				Agosti and Stewart 2007 [21]	70 BP II	Imipramine, phenelzine	No valid tool used to assess switch
				Amsterdam and Shults 2008 [17]	83 BPII	Venlafaxine	Compared to Lithium, no placebo control
				McElroy et al. 2010 (EMBOLDEN II) [14]	740 (BPI = 478, BPII = 262)	paroxetine	
				Amsterdam and Shults 2010 [15]	148 BPII	Fluoxetine	No placebo control, subsyndromal hypomania in 19.6%

*Antidepressants with mood stabilizers*							
Post et al. 2006 [40]	174	Venlafaxine	15%; no placebo control	Schaffer et al. 2006 [27]	20	Citalopram	Small sample size, no placebo control
Truman et al. 2007 [30]	366 (BPI = 240, BPII = 114)	Paroxetine, bupropion	By self-report only	Fonseca et al. 2006 [28]	20	Escitalopram	Small sample size, no placebo control
Amsterdam and Shults 2010 [15]	148 BPII	Fluoxetine	No placebo control, subsyndromal hypomania in 19.6%	Sachs et al. 2007 [29]	366 (BPI = 240, BPII = 114)	Paroxetine, bupropion	
				Perlis et al. 2010 [45]	410	Olanzapine/fluoxetine combination, lamotrigine	Conducted by Lilly Research Laboratories
				Sidor et al. 2011 [32]	1,034 (Meta- Analysis)	Fluoxetine, paroxetine, bupropion, imipramine	
